# ^18^F-FDG PET/CT in Patients with Parenchymal Changes Attributed to Radiation Pneumonitis

**DOI:** 10.4274/mirt.55706

**Published:** 2018-10-09

**Authors:** Anastas Krassenov Demirev, Irena Dimitrova Kostadinova, Iliya Rumenov Gabrovski

**Affiliations:** 1 Acıbadem City Clinic Cancer Center, Clinic of Nuclear Medicine, Sofia, Bulgaria; 2 The Queen Giovanna Hospital, Sofia, Bulgaria

**Keywords:** 18F-FDG PET/CT, radiation pneumonitis, radiotherapy, hybrid imaging

## Abstract

**Objectives:**

Radiation pneumonitis (RP) can be an adverse complication of radiotherapy (RT) and can limit the application of the already planned radiation dose. It is often associated with RT of lung carcinoma and is occasionally caused by radiation therapy of breast carcinoma and lymphomas located in the mediastinum. Positron emission tomography/computed tomography (PET/CT) emerges lately as a prospective modality for early diagnostics of RP. The aim of this study was to summarize the initial data from diagnostic application of PET/CT in patients suspicious of RP and to derive criteria, which can help differentiate RP from early recurrence of the disease and/or residual tumor.

**Methods:**

The current study included 23 patients who had metabolic (PET) and anatomical (CT) changes consistent with RP. We additionally defined metabolic activity (SUV_max_) in the lung parenchyma of 20 patients without RT.

**Results:**

All patients had increased metabolic activity in the lung parenchyma involved in the irradiated area with a mean SUV_max_ 3.45 (ranging between 1 and 7.1). The control group had a physiological background metabolic activity-SUV_max_ 0.61 +/- 0.11.

**Conclusion:**

Metabolic changes in patients suspicious of RP involved diffusely increased metabolic activity coinciding with the anatomical changes in the irradiated area. Three out of 23 patients had a proven recurrence of the primary neoplastic process in the irradiated area. The metabolic changes in those patients involved an increase in metabolic activity at follow-up or lack of tendency towards normalization after chemotherapy, which implied the existence of viable tumor cells. Our initial experience in the diagnostic application of ^18^F-FDG PET/CT in patients suspicious of RP allows us to summarize the following: PET/CT is a reliable imaging modality in the diagnostics of RP. Through its sequential use, we can differentiate inflammatory changes related to RP from early recurrence of the primary neoplastic process.

## Abstract (tr)

**Amaç:**

Radyasyon pnömonisi (RP) radyoterapinin (RT) yan etkisi olarak görülebilir ve planlanmış olan radyasyon dozunun uygulanmasını engelleyebilir. Sıklıkla akciğer karsinomu için uygulanan RT’ye bağlıdır ancak meme kanseri ve mediastinal lenfoma için uygulanan RT ile de oluşabilir. Son zamanlarda pozitron emisyon tomografi/bilgisayarlı tomografi (PET/BT), RP’nin erken tanısı için kullanılmaktadır. Bu çalışmanın amacı RP şüphesi olan hastalarda tanısal PET/BT uygulamasının ilk verilerini özetlemek ve RP’yi erken nüks ve/veya rezidü tümörden ayırt etmede kullanılabilecek kriterler oluşturmaktır.

**Yöntem:**

Bu çalışmaya RP ile uyumlu metabolik (PET) ve anatomik (BT) değişiklikleri olan 23 hasta dahil edilmiştir. Buna ek olarak RT almamış 20 hastanın akciğer parankiminde metabolik aktiviteyi (SUV_max_) değerlendirdik.

**Bulgular:**

Tüm hastalarda radyasyon uygulanmış alanlarda akciğer parankiminde metabolik aktivite artmıştı (ortalama SUV_max_ 3,45, aralık 1-7,1). Kontrol grubunda fizyolojik metabolik aktivite mevcuttu (SUV_max_ 0,61 +/- 0,11).

**Sonuç:**

RP şüphesi olan hastalardaki metabolik değişiklikler anatomik olarak radyasyon uygulanmış alanlara denk gelen diffüz artmış metabolik aktiviteyi içermekteydi. Yirmi üç hastanın üçünde bu bölgede primer neoplastik sürecin kanıtlanmış nüksü vardı. Bu hastalardaki metabolik değişiklikler arasında, canlı tümör hücrelerinin varlığını ima eder şekilde, takip sürecinde metabolik aktivitede artış ya da kemoterapi sonrası normale dönme eğiliminin olmaması bulundu. RP şüphesi olan hastalarda ^18^F-FDG PET/BT’nin tanısal uygulamasında ilk deneyimlerimiz doğrultusunda PET/BT’nin RP tanısında güvenilir bir görüntüleme yöntemi olduğu sonucuna vardık. Sürekli kullanımı ile RP’ye bağlı enflamatuvar değişiklikleri primer neoplastik sürecin erken nüksünden ayırt edebiliriz.

## Introduction

Radiation pneumonitis (RP) is an unfavorable complication that sometimes limits the course of radiotherapy (RT). It is most commonly associated with radiation therapy for lung cancer, and less frequently with other tumors such as breast cancer and mediastinal lymphoma, respectively in about 5-50%, 5-10%, and 1-5% of the cases (1,2). RP is an inflammatory reaction in the affected area of the pulmonary parenchyma. The acute stage is observed most frequently from 6 to 12 weeks after RT and symptoms include cough, shortness of breath, fever and changes in pulmonary function (3,4,5,6,7). Its chronic form occurs most often in the span of 6 to 12 months and can last up to 2 years after RT, a process associated with the development of fibrosis (8,9,10,11,12,13,14,15). Frequency and severity depend on a number of parameters, such as age, irradiated area, radiotherapeutic regimen, administered cumulative dose - most often at values above 20 Gray and almost always at doses above 40 Gray, as well as previous or concomitant chemotherapy. All of the above mentioned factors may increase drastically the effect of RT (4,11,12,15,16,17,18,19,20). Changes, attributed to RP and visualized by computed tomography (CT), are also divided into early and late ones, respectively, acute inflammatory reactions including matt glass type/infiltrative parenchymal changes and late or chronic ones (most of the cases) resulting in fibrosis (21,22). The loss of local pulmonary blood perfusion, characteristic of RP, can be visualized and quantified by conventional scintigraphy, but this method lacks sufficient specificity (23).

^18^F-FDG positron emission tomography (PET)/CT, a more recent and promising approach for early diagnostics and monitoring of patients with RP, offers a possibility for visualization of metabolic changes. Since they appear earlier than anatomical ones, detected by CT, it de facto improves the diagnostic algorithm (24,25).

The aim of this study is to summarize our initial data on the use of ^18^F-FDG PET/CT in the diagnostics of patients with parenchymal changes attributed to RP and to derive criteria for its differentiation from early recurrence, residual tumor tissue and/or metastatic lesions, thus helping us to discriminate better between inflammatory and neoplastic processes.

## Materials and Methods

This retrospective study includes 23 (n=23) patients who underwent RT in the thoracic area involving the parenchyma of the lung, and showed computer-tomographic data of RP between 2012 to 2016 in two university hospitals located in Sofia, Bulgaria. Their age range was 42-80 years (mean 62 and median 61 years). A control group comprised of 23 patients without pulmonary disease and/or neoplastic process in the thoracic area who did not undergo RT, was also evaluated. Of the patients with parenchymal and metabolic changes, 19 were women and 4 were men. Seven of them had lung cancer, 3 had Hodgkin’s lymphoma, 12 had breast carcinoma, and 1 had carcinoma of the submandibular gland and mediastinal lymphatic metastases. In 13/23 patients, serial PET/CT (pre-and post-RT) studies were performed-in 9 of the patients before and up to 6 months after RT and in 4 of the patients before and after 6 months post-RT. The remaining 10 patients underwent a single ^18^F-FDG PET/CT study up to- or over 6 months after completion of RT. The total radiation dose administered in patients suspected of RP varied between 20-60 Gray. 19/23 of the patients had chemotherapy prior to or concomitant with RT-the type of which depended on the histology, location and stage of the disease. 16/23 of the patients underwent 3D conformal RT (linear accelerator), 1 underwent intensity modulated radiation therapy (IMRT) linear accelerator and the remaining 6 patients underwent 2D conformal RT (using a Co-60 source)-data is summarized in Table 1. ^18^F-FDG PET/CT studies were conducted according to the European Association of Nuclear Medicine guidelines and included a whole body PET and CT scan performed approximately 60 minutes after intravenous injection of ^18^F-FDG with activity of up to 3 MBq/kg per patient. The CT part of the study was conducted on a 16 slice computer tomography. The quantitative accumulation of ^18^F-FDG was measured with the standardized accumulation ratio of SUV_max_.

Declaration of informed consent was signed by all patients stating that they give their full consent for their data to be used in scientific publications-above all it is a retrospective study of procedures already approved and executed.

Informed consent was obtained from each patient prior to PET/CT scanning procedure. The written document stated that the patient agrees her or his personal information as well as results from the scanning procedure be used in scientific studies and surveys.

## Results

**Figure 1:**
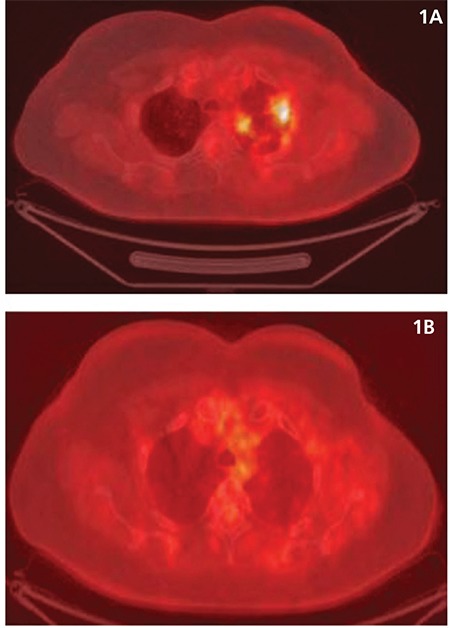
A) Metabolically active parenchymal changes located in the upper left lobe of the lung apex-the study was conducted up to 6 months after completion of radiotherapy (upper row). B) Followup study conducted 6 months after completion of radiotherapy-no significantly increased metabolic activity in the lung parenchyma along with anatomical changes that almost completely resolved-evidence of the inflammatory nature of the changes (bottom line)

**Figure 2:**
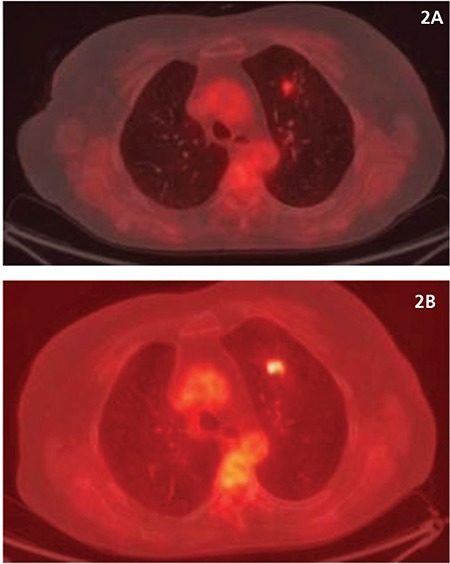
A) focal zone of increased metabolic activity in the left lobe parenchyma-visualized 14 months after completion of radiotherapy (upper row). B) shows significantly increased metabolic activity in the area seen on the previous study, the study was performed 20 months after completion of radiotherapy-subsequently, disease recurrence was diagnosed (bottom line)

All 23 patients had increased metabolic activity in the lung parenchyma involved in the RT field with a mean metabolic activity of SUV_max_ of 3.36 (+/- 1.7). Patients from the control group had physiological background metabolic activity with a mean SUV_max_ of 0.61 (+/- 0.07). In 16/23 of the patients (70%), CT changes included limited areas of consolidated lung tissue (interpreted as fibrosis). In the remaining 7/23 patients (30%) infiltrative and/or matt glass type changes were observed. Infiltrative/matt glass type CT changes were also characterized by a higher metabolic activity seen on the PET study, and were observed in patients studied up to 6 months after RT (Figure 1A, 1B). In 3/23 of the patients followed up serially with PET/CT after RT and chemotherapy, the higher metabolic activity persisted. Mean SUV_max_ remained at a mean value of 3.5 (+/- 0.8), and did not decrease (showed no trend towards decrease) to the background metabolic activity of the controls. Subsequently, those 3 cases were diagnosed with recurrence (Figure 2A, 2B).

## Discussion

According to recent studies, RP is becoming less and less frequent, mainly due to technological advances in RT and the increasing knowledge of its etiology (26,27). However, it still remains as a complication that may interfere with quality of life in cancer patients. More importantly, it can limit the application of the proper radio-therapeutic dose (28). Early and adequate diagnostics with ^18^F-FDG PET/CT hybrid imaging allows eventual modification of the RT protocol and, if necessary, initiation of an adequate therapy, in order to prevent chronic disease. On the other hand, this method also allows for visualization of early recurrence and differentiation from RP, if performed sequentially (29).

Hicks et al. (30) described the characteristic PET/CT changes in 2004, as an increased ^18^F-FDG accumulation that is the result of an active metabolic process, due to inflammatory post-radio-therapeutic changes. These changes were later characterized and quantified by Guerrero et al. (31) and defined on a scale of 0 to 3, with a linear relationship between radiation dose and metabolic activity of ^18^F-FDG in the involved lung parenchyma. However, in each of the studied patients, this metabolic response varies significantly depending on location, timing (i.e. concomitant or prior to radiation) as well as chemotherapy and RT regimen (4,20). However, these changes vary significantly between patients, depending on: location of the neoplastic process, presence of concurrent or sequential chemotherapy and type of radiation technique (4,20). The summary of the data in Table 1 is important since it gives an overview of the types of applied radiation techniques a significant part of the studied population, for example, (6/23 patients were) was treated in a 2D conformal technique with a Co-60  (Cobalt 60) teletherapy on a Co-60  unit in a 2D mode The majority of patients (16/23) was were treated with a linear accelerator in a 3D mode (conformal technique) and only one patient (1/23) underwent IMRT (3D mode-linear accelerator). The cumulative radiation dose exceeded 20 Gray in almost everyone in our patient group, a factor which contributes to the development of pulmonary injury (as stated previously) (4,9). Several studies have reported the benefits of significantly lower toxicity in the surrounding tissue after 3D radiation planning using a linear accelerator vs. 2D planning techniques (in the case of Co-60  unit) (32,33). IMRT is even superior to the previous two (2D and 3d conformal techniques) in terms of pulmonary toxicity (34). This, we consider, is one of the reasons for the higher prevalence of inflammatory and metabolically active changes involving the lung, in our relatively small group of patients. Instead of concentrating on the various reasons etiology of RP, we decided to investigate what part of those changes -1486260889 were as associated with inflammation and what part represented recurrence/metastatic spread of the main neoplastic process. After quantification of the metabolic activity in the irradiated lung and its comparison to normal pulmonary tissue, we were able to show that there is a statistically significant difference between the two (p<0.0001 - unpaired t-test). It was important to determine the physiological background metabolic activity of the lungs in order to derive criteria for the differentiation of recurrence from inflammation. In patients with confirmed disease recurrence, changes involved increased metabolic activity or lack of tendency towards normalization long after the completion of RT and chemotherapy due to the presence of vital tumor cells, a trend also observed by other authors (31). Metabolic changes attributed to pneumonitis also involved diffuse metabolic activity overlapping with the irradiated area. On the contrary, alterations consistent with recurrence were characterized by focal metabolic activity against a background of consolidated/fibrotic changes (showing no significant increase in size or anatomic change on CT images) not entirely overlapping with the involved/irradiated area of the lung. Based on our initial diagnostic experience, we recommend that all patients with increased metabolic activity in the area of the involved/irradiated volume of the lung should be followed-up by serial ^18^F-FDG PET/CT in 3 to 6 months, in order to detect early recurrence and initiate adequate and timely therapy. Several other authors also offer the same diagnostic and follow-up strategy along with verification of these findings (31).

## Conclusion

Based on our initial experience with PET/CT in patients with parenchymal changes attributed to RP, we concluded that this modality is adequate and reliable in such circumstances. Its implementation in the follow-up process can help discriminate between early recurrence of the neoplastic process and inflammatory processes.

## Figures and Tables

**Table 1:**
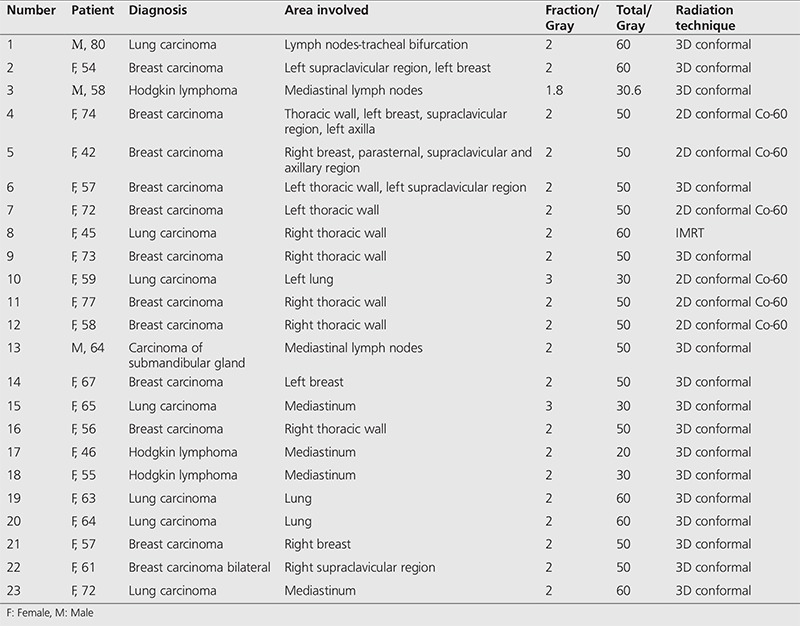
Includes patients’ age, sex, primary malignancy, area involved in radiotherapy, fraction, cumulative dose, radiation techniques used
